# Specific immune status in Parkinson’s disease at different ages of onset

**DOI:** 10.1038/s41531-021-00271-x

**Published:** 2022-01-10

**Authors:** Jun Tian, Shao-Bing Dai, Si-Si Jiang, Wen-Yi Yang, Yi-Qun Yan, Zhi-Hao Lin, Jia-Xian Dong, Yi Liu, Ran Zheng, Ying Chen, Bao-Rong Zhang, Jia-Li Pu

**Affiliations:** 1 Department of Neurology, the Second Affiliated Hospital, School of Medicine, Zhejiang University, Hangzhou, Zhejiang China; 2 Department of Anesthesiology, Women’s Hospital, School of Medicine, Zhejiang University, Hangzhou, 310009 Zhejiang China

**Keywords:** Parkinson's disease, Neuroimmunology

## Abstract

Recent evidence suggests that innate and adaptive immunity play a crucial role in Parkinson’s disease (PD). However, studies regarding specific immune cell classification in the peripheral blood in PD remain lacking. Therefore, we aimed to explore the different immune status in patients with PD at different ages of onset. We included 22 patients; among them were 10 who had early-onset PD (EOPD) and 12 had late-onset PD (LOPD) and 10 young healthy controls (YHCs) and 8 elder HCs (EHCs). Mass cytometry staining technology was used to perform accurate immunotyping of cell populations in the peripheral blood. Motor symptoms and cognitive function were assessed using the Unified Parkinson’s Disease Rating Scale (UPDRS) III score and Mini-mental State Examination (MMSE) score, respectively. *T* test and ANOVA statistical analysis were performed on the frequency of annotated cell population. Linear regression model was used to analyze the correlation between clusters and clinical symptoms. We characterized 60 cell clusters and discovered that the immune signature of PD consists of cluster changes, including decreased effector CD8^+^ T cells, lower cytotoxicity natural killer (NK) cells and increased activated monocytes in PD patients. In summary, we found that CD8^+^ T cells, NK cells, and monocytes were associated with PD. Furthermore, there may be some differences in the immune status of patients with EOPD and LOPD, suggesting differences in the pathogenesis between these groups.

## Introduction

Parkinson’s disease (PD) is the second most common neurodegenerative disorder; it is characterized by progressive dopaminergic neuron death and Lewy body, particularly α-synuclein, accumulation^1^. Recent evidence shows the significant role of neuroinflammation in PD^2^. Particularly, previous studies have correlated PD with autoimmune diseases^3^, microglia activation^4,5^, peripheral immune cell infiltration of brain histopathology^6^, and immune cell and cytokine dysregulation in the peripheral blood^7^. An aberrant immune system increases the susceptibility of developing PD; therefore, identifying pathogenic immune targets is critical to provide a new direction in exploring PD pathogenesis.

Previous studies have shown that the peripheral immune cells and molecules contribute to the development of PD through infiltration of the central nervous system (CNS) from an impaired blood–brain barrier. Notably, α-synuclein reactive T cells could even be detected even 10 years prior to PD diagnosis; this supports the role of immune inflammation in the pathogenesis of PD^8^. Dysregulation of both the innate and adaptive immune systems in the peripheral blood of patients with PD has recently been documented^9,10^. Particularly, a study found high expression levels of interleukin-4 (IL-4), IL-6, and IL-10 in patients with PD; among them, IL-6 was associated with an increased risk of mortality^11^. Similarly, the expression of cytokine chemokines is upregulated in monocytes such as monocyte chemotactic protein 1 and IL-8^12^. In addition to immune molecules, the role of peripheral immune cells in PD has received increasing attention due to the increased understanding of the impairment of blood–brain barrier in PD^13^. In the peripheral blood of PD patients, CD8^+^ T cells^6^, monocytes^14^, and NK cells^10^ are commonly increased; however, CD4^+^ T cells^15^ are decreased.

Aging is known to cause immunosenescence or the age-induced peripheral immune system dysregulation; its main feature is the accumulation of memory cells and non-functional immunocytes^16^. A recent study showed that naive CD4^+^ and naive CD8^+^ T cells were significantly decreased, whereas central memory CD4^+^ T cells were significantly increased in patients with early-stage PD^17^. They suggested that adaptive immune system altered to a more pro-inflammatory state in the EOPD patients^17^. Additionally, immune cell differentiation is diverse and complex, for example, CD8 T cells include naive CD8^+^ T cells, central memory T cells (T_CM_), effector memory T cells (T_EM_), and terminally differentiated effector memory re-expressing CD45RA T cells (T_EMRA_). Different subpopulations of the same cell type have different functions and changes, which can result in confounding results. Therefore, in this study, we aimed to explore specific immune cell subtypes in the peripheral blood in patients with PD at different ages of onset.

## Results

### Characterization of peripheral blood immune populations

**Fig. 1:**
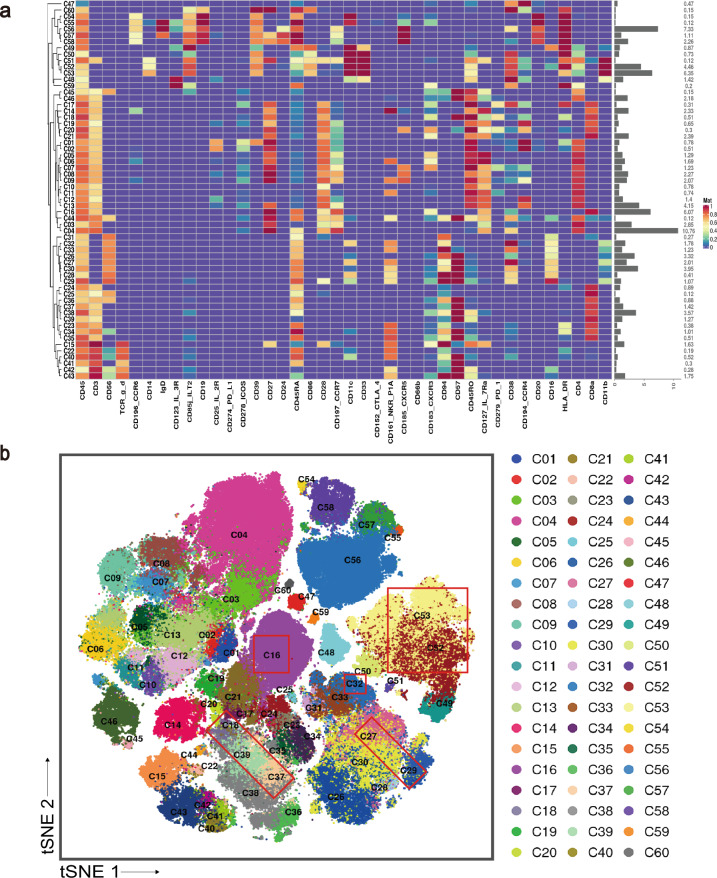
Peripheral blood immune cell clusters sorting based on marker expression levels. **a** Heatmap of normalized immune cell marker expression for 60 immune cell clusters. **b** Distribution of 60 clusters and difference in all groups. The clusters we focus on were marked with red boxes. EHCs elder healthy controls, EOPD early-onset PD, HCs healthy controls; LOPD late-onset PD, PD Parkinson’s disease, t-SNE t-distributed stochastic neighbor embedding, YHCs young healthy controls.

Mass cytometry profiling was performed in 40 peripheral blood mononuclear cell (PBMC) samples of 40 subjects; among them, 10 had EOPD and 12 had LOPD and 10 were age- and sex-matched YHCs and 8 were EHCs. All the patients we recruited were medication naive and excluded those with hypertension, diabetes, and other neurodegenerative disease. Clustering algorithm was applied to all cells, which were divided into distinct phenotypes based on marker expression^18^ (Fig. 1a). t-SNE was performed to visualize the high-dimensional data in two dimensions^19^. Distribution of each cluster (Fig. 1b) and the differences between two major groups of PD and HCs as well as among four subgroups of EOPD, LOPD, YHCs, and EHCs are displayed in Fig. 1. Unsupervised clustering was used to divide PBMCs into 60 cell clusters (C01–C60) according to the pattern of marker expression. These 60 cell clusters belonged to nine lineages, including CD4^+^ T cells, CD8^+^ T cells, γδT cells, NKT cells, NK cells, dendritic cells, monocytes and B cells. These lineages were further divided into 13 subpopulations (Supplementary Tables 1 and 2). Cell numbers were normalized to the number of PBMCs for each subject. Normalized abundances were then compared between patients with PD and HCs and among the four subgroups (EOPD, LOPD, YHCs, and EHCs). No significant difference was observed in the frequencies between total lineages except for monocytes. In contrast, significant differences were observed between several subpopulations and clusters, particularly concentrating on CD8^+^ T cells, NK cells, and monocytes.

### CD8^+^ T-cell clusters and immunosenescence in PD

**Fig. 2:**
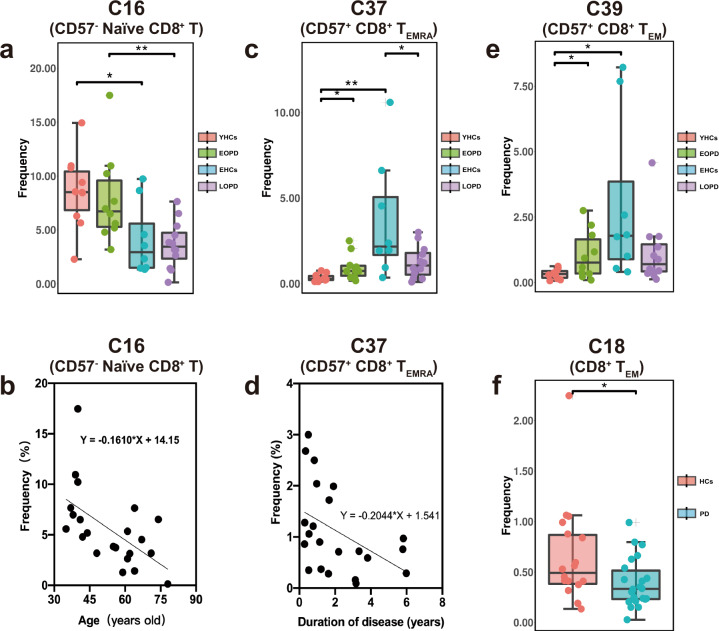
The frequency of CD8^+^ T-cell subsets between early- and late-onset PD patients and HCs and the changes of different clusters with age and course of disease. **a** The C16 of EHCs was significantly lower compared to YHCs, and was significantly lower in patients with LOPD compared to patients with EOPD. **b** The cluster of C16 cells decreased with age (*p* = 0.006, *R*^2^ = 0.323). **c** The C37 cluster of EHCs was significantly higher compared to that of YHCs. The C37 of patients with LOPD was significantly lower compared to EHCs. The C37 cluster in patients with EOPD was significantly higher compared to YHCs. **d** With the prolongation of the disease course, the C37 cells decreased (*p* = 0.037, *R*^2^ = 0.200). **e** The C39 of EHCs was significantly higher compared to YHCs. The C39 cluster of patients with EOPD was significantly higher compared to YHCs. **f** The frequency of C18 of PD patients was significantly lower compared to HCs. Two-sided *t* tests were used to test statistical significance between groups (*p,0.05, **p,0.01, ***p,0.001). Error bars show the mean ± SEM. EHCs elder healthy controls, EOPD early-onset PD, HCs healthy controls; LOPD late-onset PD, PD Parkinson’s disease, YHCs young healthy controls.

Among 14 clusters of CD8^+^ T cells, several clusters were significantly differed between groups or subgroups. The frequency of C16 (CD57^−^ naive CD8^+^ T, Fig. 2a) was lower in EOPD than in LOPD; particularly, an inverse relationship was observed between age and C16 frequency (Fig. 2b). Moreover, C16 was also decreased in EHCs compared with YHCs (Fig. 2a) although no statistical difference was noted using ANOVA with Bonferroni correction (Supplementary Fig. 2a). C18 (CD8^+^ T_EM_), a type of CD45RA^−^ CCR7^−^ effector memory T cells, was significantly decreased in the PD group than in the HC group (Fig. 2f). C37 belonged to CD57^+^ CD8^+^ T_EMRA_, a type of CD45RA^+^ CCR7^−^ effector memory re-expressing CD45RA T cells, and increased with age in HCs (Fig. 2c). Compared with the EHCs, C37 was decreased in patients with LOPD (Fig. 2c) although no statistical difference was observed using ANOVA (Supplementary Fig. 2c). Additionally, C37 decreased in patients with prolonged PD duration (Fig. 2d). CD8^+^ T_EMRA_ and T_EM_ were effector CD8^+^ T cells and CD8^+^ T_EM_ cells required repeated proliferation to transform into CD8^+^ T_EMRA_ cells, which was considered to be terminally differentiated with high cell killing capacity, increased perforin and granzyme B releasing^20^.

Additionally, we speculated that there were several differences between patients with EOPD and LOPD. The frequency of C16 was lower in EOPD than in LOPD (Fig. 2a). C37 was higher in patients with EOPD than in YHCs and lower in patients with LOPD than in EHCs (Fig. 2c). The trend was similar to C39 (Fig. 2e), though there were no significant difference was noted using ANOVA (Supplementary Fig. 2c, e).

### Toxicity of NK cells in PD

**Fig. 3:**
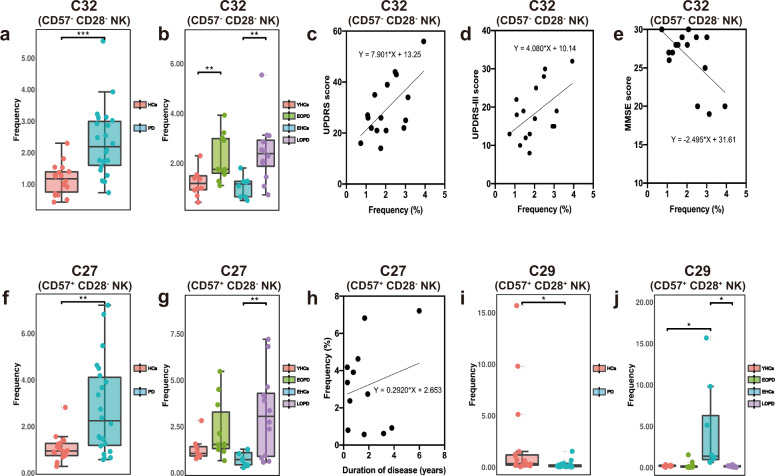
The frequency of NK cell clusters between PD patients and HCs and the changes of different clusters with disease course, UPDRS, UPDRS-III, and MMSE scores. **a** The frequency of C32 subgroup in patients with PD was significantly higher compared to HCs. **b** The C32 cluster in patients with LOPD was significantly higher compared to EHCs and was significantly higher in patients with EOPD compared to YHCs. **c** The increase in C32 frequency was associated with an increase in the UPDRS score (*p* = 0.012, *R*^2^ = 0.375). **d** The UPDRS-III score increased (*p* = 0.046, *R*^*2*^ = 0.254). **e** The MMSE score decreased (*p* = 0.013, *R*^2^ = 0.367). **f** C27 in patients with PD was significantly higher compared to HCs. **g** C27 in patients with LOPD was significantly higher compared to EHCs. **h** Prolonged disease course was associated with increased C27 cell clusters in patients with LOPD (*p* = 0.041, *R*^*2*^ = 0.424). **i** C29 in patients with PD was significantly lower compared to HCs. **j** C29 in patients with LOPD was significantly lower compared to YHCs. (*p,0.05, **p,0.01, ***p,0.001). Error bars show the mean ± SEM. EHCs elder healthy controls, EOPD early-onset PD, HCs healthy controls, LOPD late-onset PD, PD Parkinson’s disease, YHCs young healthy controls.

Three clusters (C27, C29, and C32) of NK cells were significantly different between groups and subgroups. Among them, C32 (CD56^+^, CD16^+^, CD57^−^, CD28^−^) and C27 (CD56^+^, CD16^+^, CD57^+^, CD28^−^) increased in PD patients compared to those in HCs, and both increased in patients with LOPD compared to those in EHCs (Fig. 3a, b, f, g). In contrast to C27 and C32, the C29 (CD56^+^, CD16^+^, CD57^+^, CD28^+^) cluster was lower in patients with PD (Fig. 3i, j), although no significant difference was observed using ANOVA between the four subgroups in C29 (Supplementary Fig. 3j).

Apparently, three clusters of NK cells differ in their surface expression of CD28 and CD57. The presence of CD28 has been considered a strong activator of NK cells, which induces degranulation, lysis of target cells and production of pro-inflammatory cytokines^21^. In addition, NK cell expression of CD57 could be considered a more mature phenotype, higher cytotoxic capacity, and more sensitive to CD16 stimulation^22^. From the expression of CD28 and CD57, we speculate that the cytotoxicity of C29 cells were the highest, followed by that of C27 cells and C32 cells. We found that the increase in CD57^−^ CD28^−^ NK cells (C32) were associated with increased UPDRS and UPDRS-III scores (Fig. 3c, d) and decreased MMSE scores (Fig. 3e). Additionally, an increase in the CD57^+^ CD28^−^ NK cells (C27) of patients with LOPD were observed as the disease prolonged (Fig. 3h). In conclusion, the peripheral blood of patients with PD demonstrated a decrease in highly toxic NK cells and an increase in less toxic NK cells. In addition to differences in immunological background, the NK cell changes between EOPD and LOPD were broadly similar. Patients with PD and LOPD had relatively consistent immune trends, which may be attributed to the large number of patients with LOPD.

### Activated monocytes in PD

**Fig. 4:**
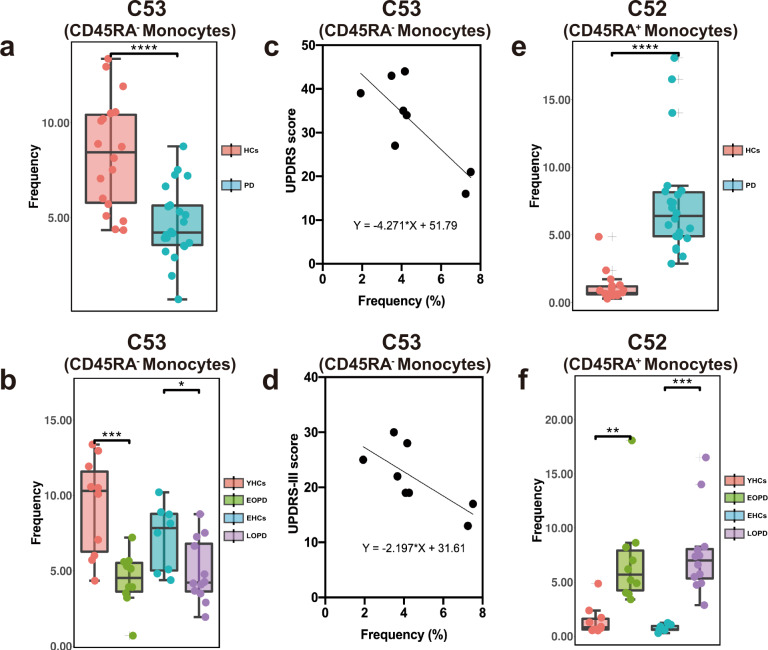
The frequency of monocyte clusters between PD patients and HCs, and the changes in different clusters with UPDRS and UPDRS-III scores. **a** The frequency of C53 in patients with PD was significantly lower compared to HCs. **b** The C53 cluster in patients with EOPD was significantly lower compared to YHCs; additionally, C53 in patients with LOPD was significantly lower compared to EHCs. **c** Increased C53 frequency was associated with an increased UPDRS score (*p* = 0.018, *R*^2^ = 0.633) and **d** UPDRS-III scores increased in patients with LOPD (*p* = 0.042, *R*^2^ = 0.526). **e** The proportion of the C52 subgroup in patients with PD was significantly higher compared to HCs. **f** C52 in EOPD patients was significantly higher compared to YHCs and was significantly higher in patients with LOPD compared to EHCs (*p,0.05, **p,0.01, ***p,0.001). Error bars show the mean ± SEM. EHCs elder healthy controls, EOPD early-onset PD, HCs healthy controls, LOPD late-onset PD, PD Parkinson’s disease, YHCs young healthy controls.

Finally, we found that all monocyte clusters (C53 and C52) were different between groups and subgroups. C53 (CD14^+^, CD16^−^, CD45RA^−^) decreased in PD compared to HCs (Fig. 4a), which was similarly found in the LOPD subgroup (Fig. 4b) and not in the EOPD subgroups using ANOVA (Supplementary Fig. 4b). By contrast, C52 (CD14^+^, CD16^−^, CD45RA^+^) increased in PD (Fig. 4e), and a consistent trend was found in both the late- and early-onset patients (Fig. 4f).

CD45RA was originally thought to be a marker for the naive T cells. It was later found that CD45RA^+^ T cells accumulated in vivo due to persistent viral infection, inflammatory syndromes and senescence^23^. Furthermore, several studies have also found that CD45RA was expressed on activated monocytes, and was regarded as a marker of peripheral blood monocyte activation^24^. We speculate that in patients with EOPD, the proportion of inactivated monocytes (C53) decreased and the proportion of activated monocytes (C52) increased. Regarding monocytes, the two cells (C52 and C53) presented similar tendencies in EOPD and LOPD. Interestingly, the decrease in C53 was more pronounced in early-onset PD, whereas this change was not statistically different in late-onset PD (Supplementary Fig. 4b). In general, we found that the activation of monocytes was the common manifestation of the PBMCs in patients with PD.

## Discussion

In this study, we identified that PD was closely associated with specific immune cell classification, such as clusters C16, C18 and C37 of CD8^+^ T cells; clusters C32, C27 and C29 of NK cells; and clusters C53 and C52 of monocytes in the peripheral blood. These findings suggest the difference in the immune background in PD.

Aging is known to cause gradual weakening of body function, including the immune system^25^; this presents with abnormal immune responses and senescence phenotype of immune cells. Thus, aging causes a decrease in CD57^−^ naive CD8^+^ cells and an increase in CD57^+^ CD8^+^ effector T cells^26,27^. CD57 expression is usually associated with senescent human CD8^+^ T cells and represents “end-stage” effector T cells that are unable to proliferate^28^. However, it has also been shown that CD57^+^ CD8^+^ effector T cells may be capable of rapid cytokinesis, cytotoxicity and IFN-γ production^29^. Interestingly, we found significantly lower numbers of CD8^+^ effector T cells in patients with PD compared to controls, despite the importance of senescence in the exacerbation of neurodegenerative disorders such as PD. Moreover, recent studies have shown a decrease in markers of replicative senescence in the CD8^+^ cells of PD patients, such as CD57 and T_EMRA_ cells, which is consistent with our findings^27^. This may lead to insufficient antigen processing in T cells, which results in autoantigen accumulation, such as α-synuclein. In contrast, the decreased CD8^+^ effector T cells in peripheral blood may be due to transfer to the brain. There is research evidence of tissue-resident memory CD8^+^ T cells in the postmortem brain tissue of patients with PD, which were associated with synucleinopathy and neuronal death^6^. In mouse models, CD8^+^ T infiltration was similarly found in the brain, which activated and converted microglia to the M1 pro-inflammatory phenotype^30^.

NK cells are important in the field of oncology as a member of the innate immune system^31^. NK cells have recently been found to play different roles in adaptive immune regulation. Chemokines produced by neurons, microglia, and astrocytes can recruit peripheral NK cells to the CNS, which effectively clears α-synuclein aggregates through receptor-mediated endocytosis^32^. Additionally, NK cells were found in the substantia nigra of both humans^33^ and mice^34^; this is supported by the association found between NK cells and increased phosphorylated α-synuclein deposits in mouse models of synucleinopathies^33^. Previous studies have found that the number of circulating NK cells in patients with PD is increased compared to that in controls; additionally, their activity is associated with disease severity^35^. We found that CD57^−^, CD28^−^ and CD57^+^, CD28^−^ NK cells were increased and CD57^+^, CD28^+^ NK cells were decreased in PD patients compared with those in HCs. Both CD28 and CD57 are associated with the cytotoxicity of NK cells. NK cells require CD28-mediated costimulatory signals for optimal proliferation and are able to produce high levels of interferon (IFN)-γ and tumor necrosis factor (TNF) in response to CD28 antibody stimulation^36^. Additionally, CD57 antigen can be considered as a marker of terminal differentiation in NK cells and is strongly associated with inflammation^37^. CD28^−^ and CD57^−^ NK cells may result in impaired antigen clearance and immunoinflammatory disorders in PD. Further, recent studies have shown that NK cell depletion resulted in exacerbated synuclein pathology in the mouse model, suggesting the protective role of NK cells in PD^33^. This was consistent with our findings that NK cells were correlated with the severity of the disease because increase in C32 NK cells were associated with increased UPDRS and UPDRS-III scores, but decreased MMSE scores of PD patients. Therefore, we speculated that impaired NK cell function was a sign of poor prognosis. The exact mechanism of the protective role of NK cells remains unknown; however, astrocytes were recently found to suppress CNS inflammation by triggering T-cell apoptosis. This process was driven by the production of IFN-γ from meningeal NK cells, the expression of which is regulated by gut microbiome^38^.

Studies have found that the chemokines released by microglia or the expression of full-length human α-synuclein allows the peripheral pro-inflammatory monocyte infiltration of the substantia nigra^39^. The relative number of monocytes was found to be significantly higher in the prefrontal cortex of patients with PD than in those of normal controls^14^. Activated monocytes can infiltrate the brain parenchyma through similar mechanisms and kill neurons. Additionally, they may recognize and promote the transmission of peripheral antigens, such as α-synuclein^14^. Previous studies have similarly shown monocyte activation in the periphery of PD. Patients with PD had significantly higher proliferative capacity of monocytes compared to controls, which was associated with a shorter disease duration and later onset^40^. Another study concluded that monocyte phagocytosis was higher in early-moderate PD and the changes in monocytes were mainly attributed to the autologous serum^41^. Similarly, it has been shown that the inflammation susceptibility of the monocytes in PD may be attributed to the “second strike”, that was the lipopolysaccharide (LPS)- and α-synuclein protein-induced inflammation^42^. LPS is a microbial metabolite that has been shown to promote the entry of monocytes into the CNS causing different disorders^42^.

The phenotype of immune cells from elderly individuals showed significant changes. Particularly, thymic degeneration and repeated pathogen exposure caused a decrease in naive CD8^+^ T cells and an accumulation in mutual memory cells, specifically terminally differentiated effector CD8^+^ T cells, which is consistent with our findings. Additionally, CD8^+^ T cells and NK cells entering a senescent state are characterized by the absence of CD28 expression and increased expression of CD57. Aged immune cells can produce several specific substances, such as chemokines and cytokines, leading to a pro-inflammatory environment in the body^43^. However, immunosenescence has been reported to play a protective role against autoimmune diseases such as PD due to the decreased autoantigen response of non-functioning immune cells^44^. By contrast, stronger autoimmune responses may be triggered in patients with EOPD. These conflicting findings show the complex circumstance of immune aging in LOPD and EOPD. Indeed, we found the difference of peripheral blood immune cells between EOPD and LOPD such as CD8^+^ T_EMRA_ and CD45RA^−^ monocytes.

Peripheral blood immune status is relatively heterogeneous in different individuals and could be influenced by various factors such as medicines, underlying diseases, mental^45^, diet^46^, exercise^46^, etc. Studies have shown that a variety of PD medicines have the effect on immune cell phenotype and function. Dopamine is a significant modulator of immune function and can affect a wide range of immune cells, which express almost all dopamine receptors^47^. Among leukocytes, B cells and NK cells have the highest expression of dopamine receptors^47^. In the experimental autoimmune encephalomyelitis mice, pramipexole inhibited the production of inflammatory cytokines such as IL-17, IL-1β and TNF-α in peripheral lymphoid tissues^48^. In addition, selegiline changed the phagocytic activity of granulocytes in the mice, which caused a decrease in B cells and an increase in T lymphocytes, especially CD8^+^ T cells in the spleen^49^. Overall, the interference from PD drugs should be carefully considered when studying immune status in PD.

Our study has some limitations. First, the relatively small number of patients involved in the study may have caused bias in the results. Second, cytomegalovirus antibody levels were not evaluated, which are associated with CD8^+^ T-cell expression^50^. Third, our study has not further validated the function of the immune cells through cellular or animal experiments. In the next studies we will select the interested clusters to explore more deeply the mechanisms of changes in peripheral immune cells in PD. Furthermore, future studies with a larger sample size are warranted to consider these contributing factors and validate the relevance of NK cells and monocytes in disease progression to further explore the pathogenesis between immunity and PD.

In summary, our data reflected the changes in peripheral blood immune cells in patients with PD. Particularly, we found that CD8^+^ T cells, NK cells, and monocytes were changed and part of the clusters were associated with age of onset, disease duration, or scores of motor or psychiatric symptoms of PD, which provides an evidence for the role of neuroinflammation in PD. Additionally, we found differences regarding the changes of peripheral cells between EOPD and LOPD. This finding suggests immunological specificities and highlights the need for new explanations regarding the role of neuroinflammation in EOPD.

## Methods

### Subjects

Our study included 22 patients with PD. Among them, 10 had early-onset PD (EOPD; <50 years old) and 12 had late-onset PD (LOPD; >50 years old). All patients were admitted to the neurology department at the Second Affiliated Hospital of Zhejiang University and diagnosed by senior movement disorder specialists based on the current diagnostic criteria^51^. Additionally, 10 age- and sex-matched YHCs and 8 EHCs were recruited (Table 1 and Supplementary Table 4). Patients with hypertension, diabetes, and other neurodegenerative diseases, including essential tremor, multiple-system atrophy, corticobasal degeneration, and Wilson’s disease, were excluded. All the patients we recruited were medication naive. Data regarding the age of onset, duration of disease, Hoehn and Yahr (H&Y) stage, and UPDRS and MMSE scores were collected (Table 1), and the peripheral blood samples were obtained.

**Table 1:** Demographic characteristics of PD patients and healthy controls.

	YHCs	EOPD	EHCs	LOPD
Total participants enrolled, *n*	10	10	8	12
Age (Mean)	40.30	40.30	68.25	64.23
Gender (Female/Male)	1/9	3/7	4/4	4/5
Age of onset (Mean)	N/A	37.8	N/A	62.42***
Duration (Mean)	N/A	2.44	N/A	1.8
UPDRS score (Mean)	N/A	26.5	N/A	32.38
UPDRS-III score (Mean)	N/A	15.38	N/A	21.63
H-Y stage (Mean)	N/A	1.31	N/A	1.44
MMSE score (Mean)	N/A	26.63	N/A	26.38

Two-sided t-test was used to test statistical significance between groups.

*EHCs* elder healthy controls, *EOPD* early-onset PD, *LOPD* late-onset PD, *YHCs* young healthy controls.

***p,0.001.

### Standard protocol approvals, registrations, and patient consents

Ethics approval was obtained through the Medical Ethics Committee of the Second Affiliated Hospital of Zhejiang University School of Medicine (2020–596). All patients and HCs provided their informed consent before blood withdrawal.

### Sample processing for mass cytometry

The blood samples were transferred to a 50-ml centrifuge tube with a 10-ml pipette. Ficoll separation solution (GE Healthcare) at 10 ml was added to the 50-ml centrifuge tube. The sample was diluted to 20 ml with PBS (GENOM) and was added slowly to the top layer of Ficoll separation solution without breaking the upper liquid level of the Ficoll separation solution. The 50-ml centrifuge tube containing Ficoll and sample was placed in a centrifuge with plate rotor (Avanti J-15R, Beckman). Then, it was centrifuged at 400 × *g* for 15 min (Acc/Dec Rate 1). The waste liquid above the separated white film layer was absorbed with a suction pump, and 0.5 ml volume was reserved to prevent PBMC loss. The white layer was transferred to a new 50-ml centrifuge tube using a 1-ml manual pipette, and repeated aspiration was performed until no obvious cells remained in the Ficoll layer. FACS buffer was added to PBMC initial extract to supplement to 30 ml, followed by 400 × *g* centrifugation for 10 min to adsorb and discard the supernatant. A 1-ml ACK (PLT) fine was added. Cell lysis was done; then, it was blown, mixed, and let stand for 2 min. FACS buffer was added into PBMC suspension after cracking to complement to 10 ml. It was centrifuged at 400 × *g* for 5 min. The supernatant was discarded, and 4-ml FACS buffer was added and blown and resuspend. A 10-μl cell suspension was obtained, and Trypan blue dye (Solarbio) was added and diluted to count. The supernatant was discarded by centrifugation and cell precipitation was obtained.

### Mass cytometry staining and data acquisition

Cells were washed once with 1× PBS; to exclude dead cells, they were stained with 100 μl of 250 nM cisplatin (Fluidigm) on ice for 5 min. Afterwards, they were incubated in Fc receptor blocking solution before staining with surface antibodies cocktail for 30 min on ice (Supplementary Table 3). Cells were washed twice with FACS buffer (1× PBS + 0.5% BSA) and fixed in 200 μl of intercalation solution (Maxpar Fix and Perm Buffer containing 250 nM 191/193 Ir, Fluidigm) overnight. After fixation, cells were washed once with the FACS buffer and then a perm buffer (eBioscience); then, cells were stained with intracellular antibodies cocktail for 30 min on ice (Supplementary Table 3). Cells were washed and resuspended with deionized water, adding to 20% EQ beads (Fluidigm), acquired on a mass cytometer (Helios, Fluidigm).

### CyTOF data analysis

Data of each sample were de-barcoded from raw data with unique mass-tagged barcodes using a doublet-filtering scheme^18^. Bead normalization method was used to normalize the fcs file generated from different batches^52^. Data were manually gated using the FlowJo software to exclude debris, dead cells, and doublets, leaving live, single immune cells. The X-shift clustering algorithm was applied^53^ to all cells to partition the cells into distinct phenotypes based on their marker expression levels. Cell types of each cluster were annotated according to the marker expression pattern on a heatmap of cluster versus marker. The dimensionality reduction algorithm, t-distributed stochastic neighbor embedding (t-SNE), was used to visualize the high-dimensional data in two dimensions and to show the distribution of each cluster and marker expression and differences among each group or different sample types^54^.

### Statistical analyses

The differences in the frequency of each cluster between patients with PD and HCs were compared. Additionally, the differences in the frequency of each cluster between YHCs and EHCs, EOPD patients and YHCs, LOPD patients and EHCs, and EOPD patients and LOPD patients were compared. Two-sided *t* test statistical analysis was used to test the statistically significant difference between groups and subgroups. Additionally, we also performed one-way ANOVA analysis among all four subgroups (YHCs, EHCs, EOPD and LOPD) and Bonferroni correction was used as multiple comparison adjustment. A linear regression model was used to analyze the correlation between clusters and clinical characteristics (age, duration of disease, UPDRS score, UPDRS-III score, H&Y stage, and MMSE score) of patients with PD. All statistical analyses were performed using SPSS version 25.0 (Armonk, NY: IBM Corp). Statistical significance was set at *p* < 0.05.

### Reporting summary

Further information on research design is available in the Nature Research Reporting Summary linked to this article.

## Supplementary information

Supplementary Files

Reporting Summary

## Data Availability

Anonymized data not published within this article will be made available on request from any qualified investigator.
