# Supplementary material for: Specific immune status in Parkinson’s disease at different ages of onset
Source: NPJ Parkinsons Dis. 2022 Jan 10;8:5. doi: 10.1038/s41531-021-00271-x (PMC8748464; doi:10.1038/s41531-021-00271-x)
Supplement: Supplementary file 1 — Supplementary Files [file 41531_2021_271_MOESM1_ESM.pdf]

Supplementary Figures with legends beneath each one

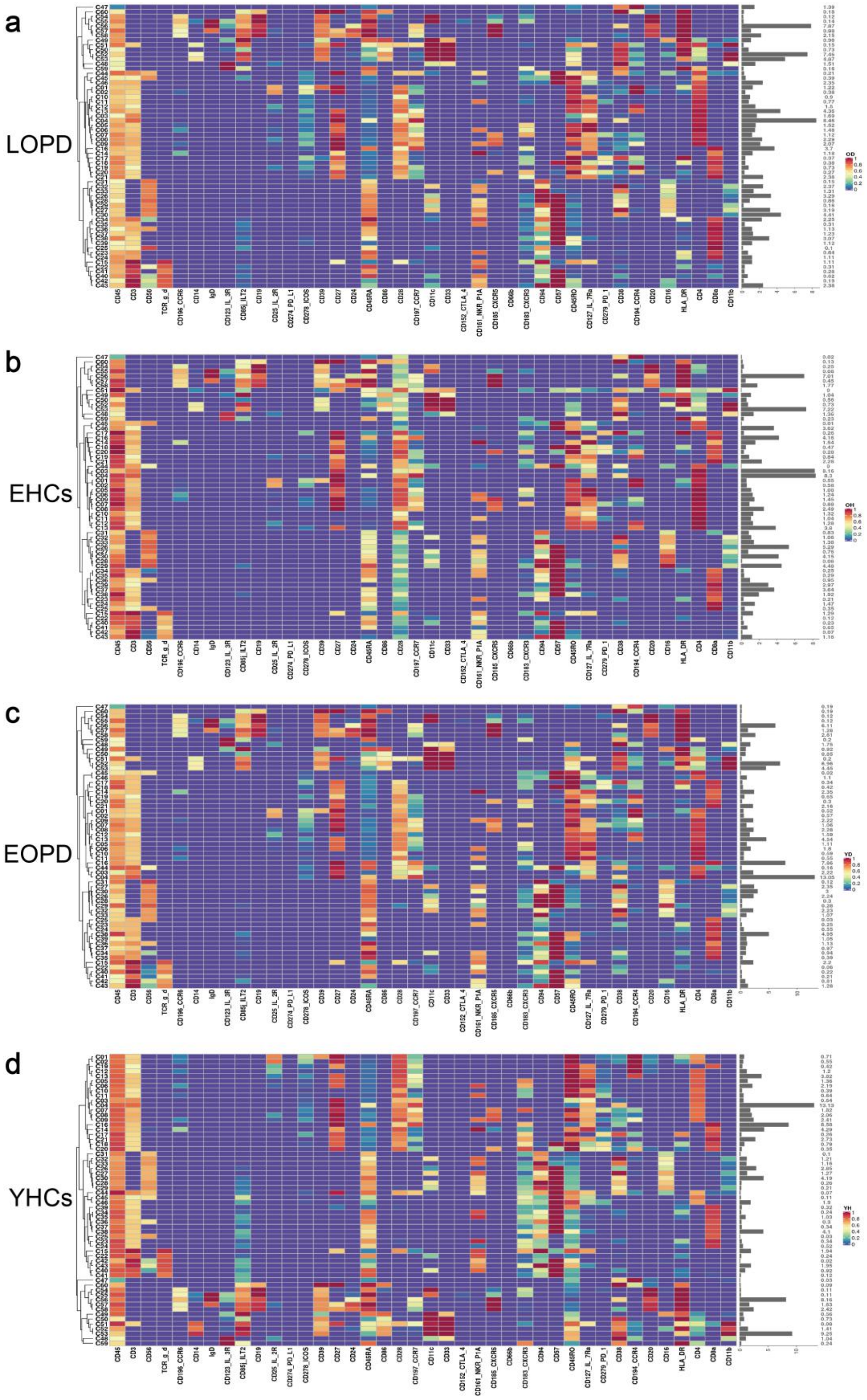

Supplementary Fig. 1 The phenotype of peripheral blood immune cell clusters in late-onset PD, elder healthy controls, early-onset PD and young healthy controls.

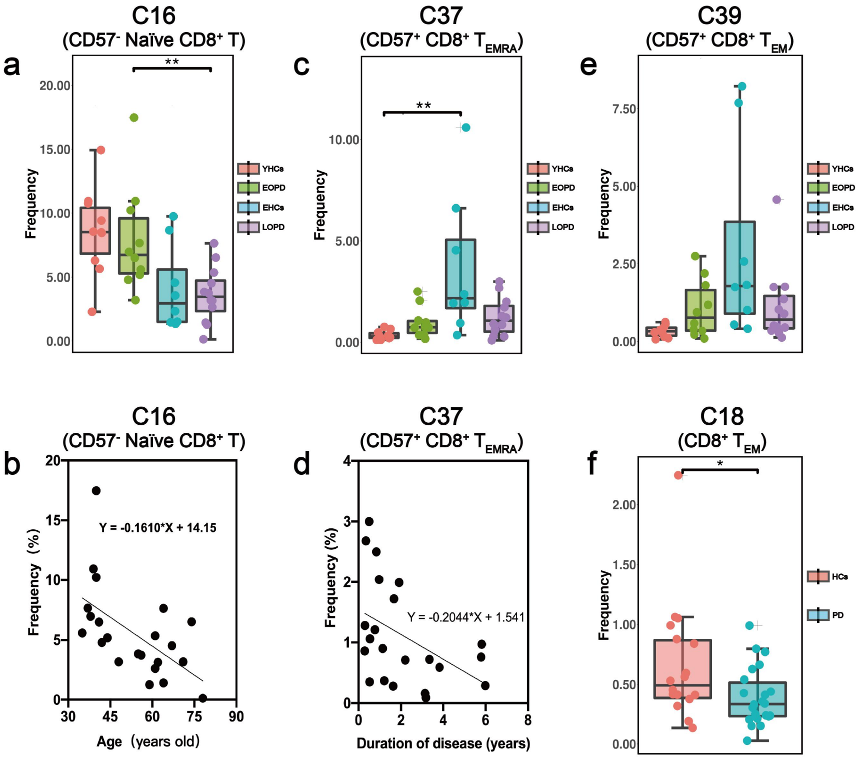

Supplementary Fig. 2 The frequency of CD8$^+$ T cell subsets between early and late onset PD patients and HCs using ANOVA with Bonferroni correction. (**a**) The C16 was significantly lower in patients with LOPD compared to patients with EOPD. (**b**) The cluster of C16 cells decreased with age (p = 0.006, R^2 = 0.323). (**c**) The C37 cluster of EHCs was significantly higher compared to that of YHCs. (**d**) With the prolongation of the disease course, the C37 cells decreased (p = 0.037, R^2 = 0.200). (**e**) The C39 of EHCs was higher compared to YHCs although there was no statistical difference. (**f**) The frequency of C18 of PD patients was significantly lower compared to HCs. ANOVA with Bonferroni correction was used to test statistical significance between groups (*p,0.05, **p,0.01, ***p,0.001). Error bars show the mean ± SEM. EHCs = elder healthy controls; EOPD = early-onset PD; HCs = healthy controls; LOPD = late-onset PD; PD = Parkinson’s disease; YHCs = young healthy controls.

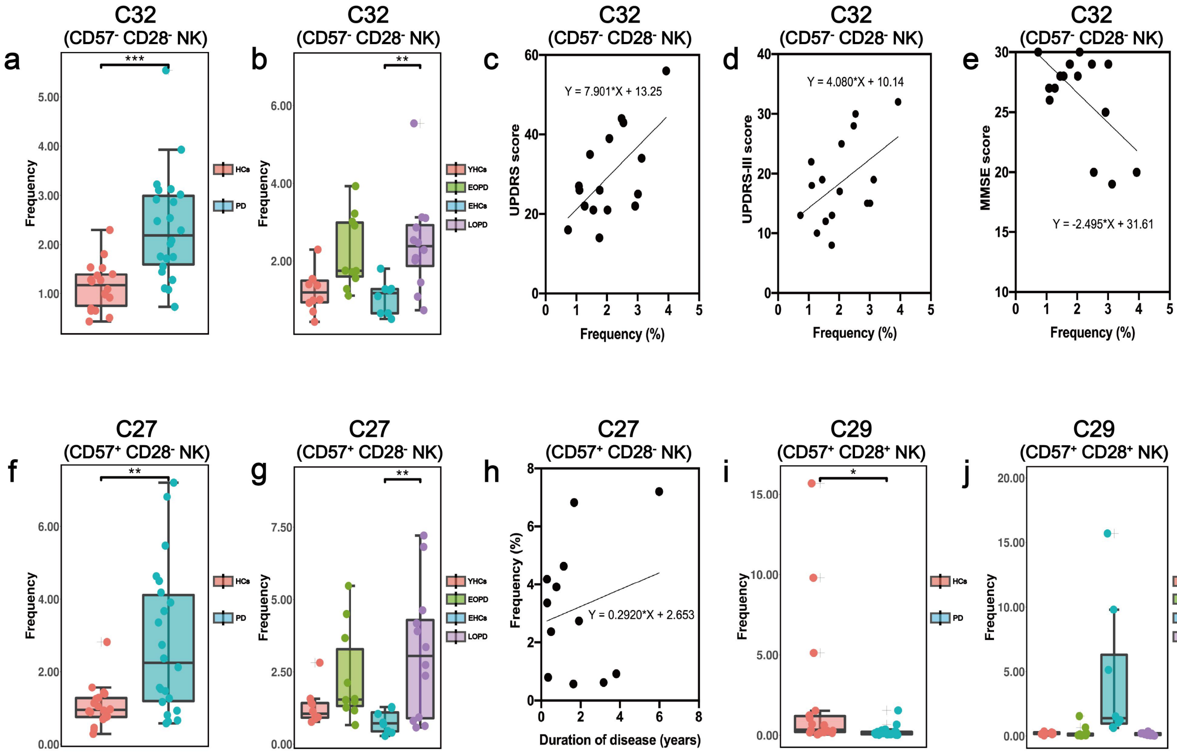

Supplementary Fig. 3 The frequency of NK cell clusters between PD patients and HCs using ANOVA with Bonferroni correction. (**a**) The frequency of C32 subgroup in patients with PD was significantly higher compared to HCs. (**b**) The C32 cluster in patients with LOPD was significantly higher compared to EHCs. (**c**) The increase in C32 frequency was associated with an increase in the UPDRS score (p = 0.012, R^2 = 0.375). (**d**) The UPDRS-III score increased (p = 0.046, R^2 = 0.254). (**e**) The MMSE score decreased (p = 0.013, R^2 = 0.367). (**f**) C27 in patients with PD was significantly higher compared to HCs. (**g**) C27 in patients with LOPD was significantly higher compared to EHCs. (**h**) Prolonged disease course was associated with increased C27 cell clusters in patients with LOPD (p = 0.041, R^2 = 0.424). (**i**) C29 in patients with PD was significantly lower compared to HCs. (**j**) C29 in patients with LOPD was lower compared to YHCs although there was no statistical difference. ANOVA with Bonferroni correction was used to test statistical significance between groups (*p,0.05, **p,0.01, ***p,0.001). Error bars show the mean ± SEM. EHCs = elder healthy controls; EOPD = early-onset PD; HCs = healthy controls; LOPD = late-onset PD; PD = Parkinson's disease; YHCs = young healthy controls.

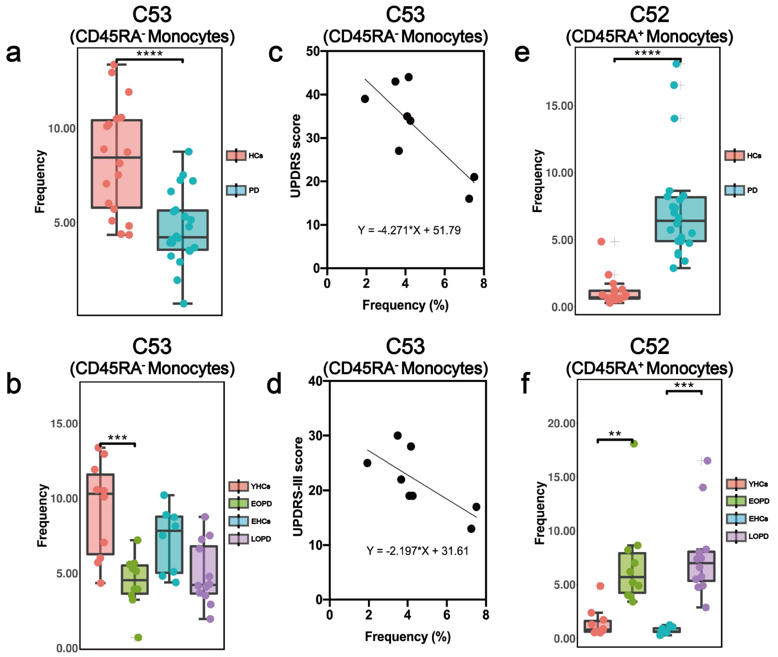

Supplementary Fig. 4 The frequency of monocyte clusters between PD patients and HCs using ANOVA with Bonferroni correction. (**a**) The frequency of C53 in patients with PD was significantly lower compared to HCs. (**b**) The C53 cluster in patients with EOPD was significantly lower compared to YHCs; additionally, C53 in patients with LOPD was lower compared to EHCs although there was no statistical difference. (**c**) Increased C53 frequency was associated with an increased UPDRS score (p = 0.018, R^2 = 0.633) and (**d**) UPDRS-III scores increased in patients with LOPD (p = 0.042, R^2 = 0.526). (**e**) The proportion of the C52 subgroup in patients with PD was significantly higher compared to HCs. (**f**) C52 in EOPD patients was significantly higher compared to YHCs and was significantly higher in patients with LOPD compared to EHCs. ANOVA with Bonferroni correction was used to test statistical significance between groups (*p,0.05, **p,0.01, ***p,0.001). Error bars show the mean ± SEM. EHCs = elder healthy controls; EOPD = early-onset PD; HCs = healthy controls; LOPD = late-onset PD; PD = Parkinson's disease; YHCs = young healthy controls.

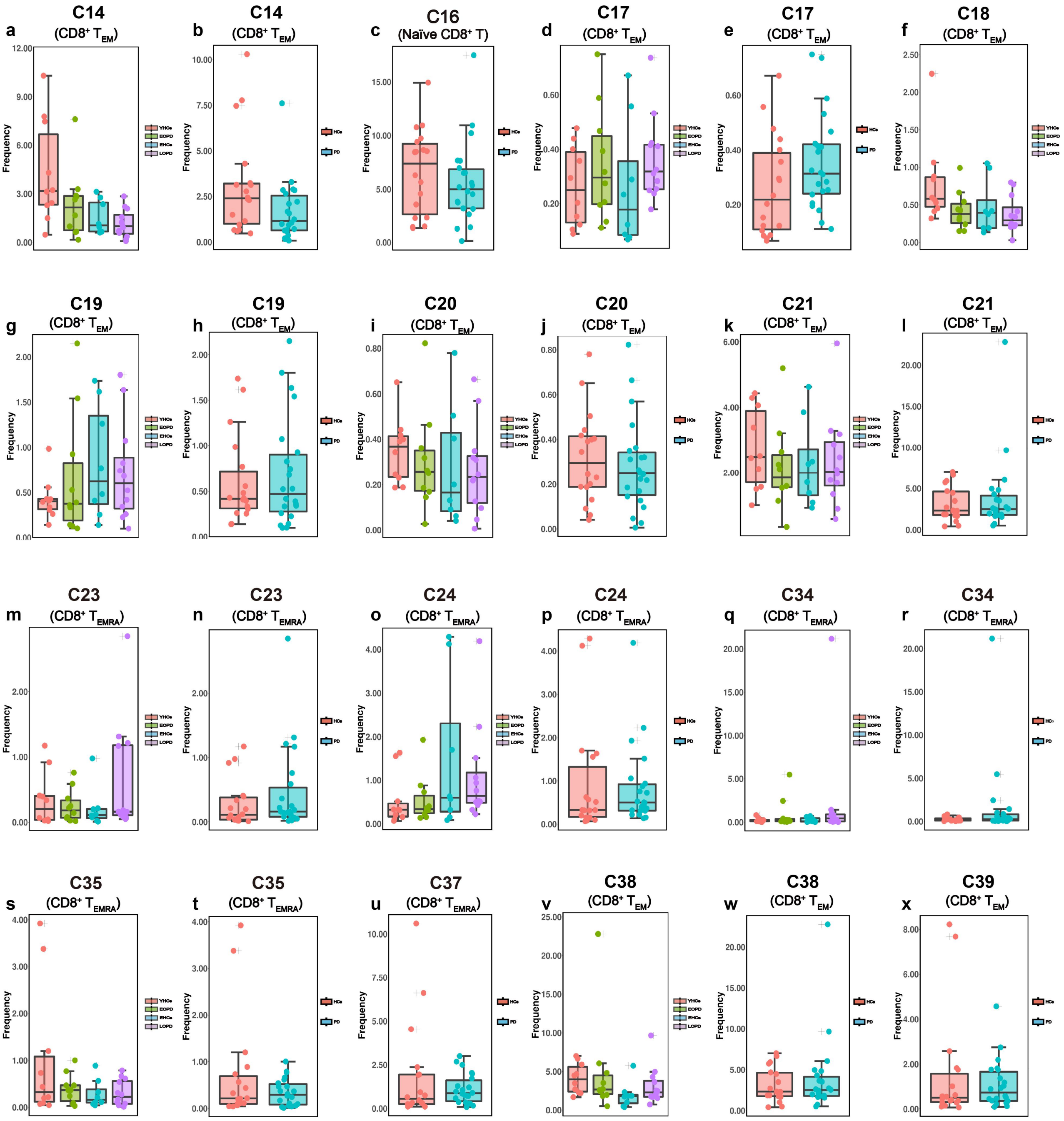

Supplementary Fig. 5 Other clusters of CD8$^+$ T cells between early and late onset PD patients and HCs using ANOVA with Bonferroni correction. ANOVA with Bonferroni correction was used to test statistical significance between groups (**p*,0.05, ***p*,0.01, ****p*,0.001). Error bars show the mean ± SEM. EHCs = elder healthy controls; EOPD = early-onset PD; HCs = healthy controls; LOPD = late-onset PD; PD = Parkinson’s disease; YHCs = young healthy controls.

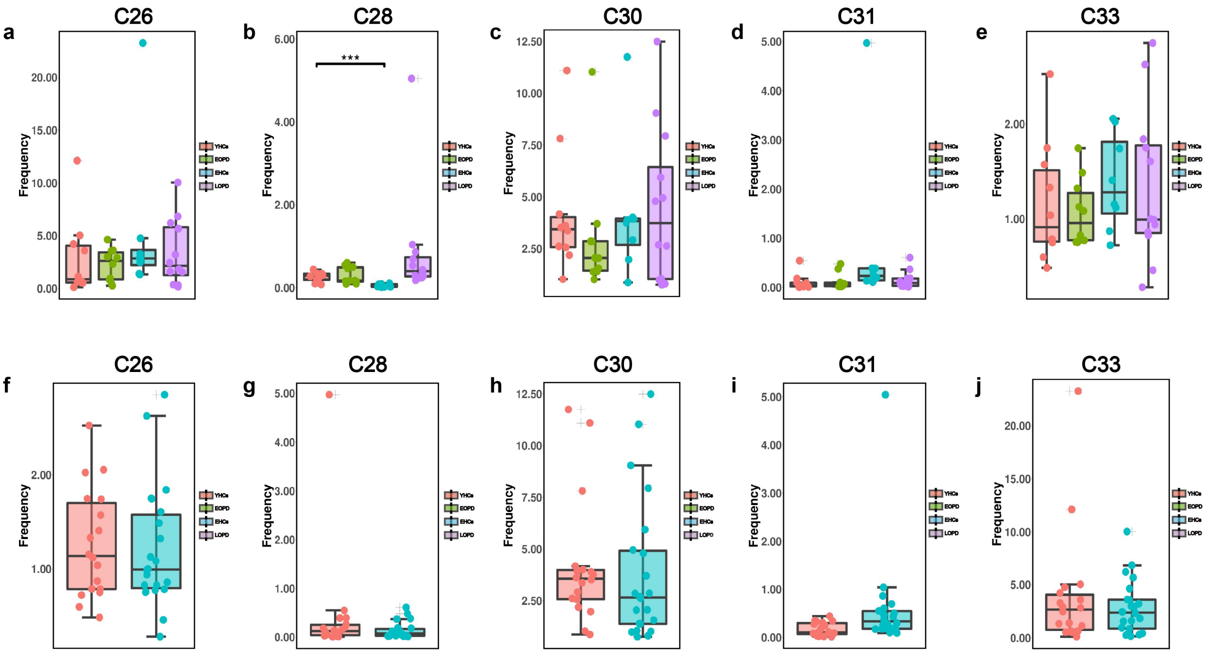

Supplementary Fig. 6 Other clusters of NK cells between early and late onset PD patients and HCs using ANOVA with Bonferroni correction. ANOVA with Bonferroni correction was used to test statistical significance between groups (**p*,0.05, ***p*,0.01, ****p*,0.001). Error bars show the mean ± SEM. EHCs = elder healthy controls; EOPD = early-onset PD; HCs = healthy controls; LOPD = late-onset PD; PD = Parkinson's disease; YHCs = young healthy controls.

Supplementary Tables

Supplementary Table 1: List of lineages and subpopulations derived from PBMCs

Lineage	Subpopulation	Cluster	Markers
$CD4^+$ T cell	Naïve $CD4^+$ T	C03- C04	$CD3^+$ $CD4^+$ $CD8^-$ $CD45RO^-$ $CCR7^+$
	T_{CM}	C01-C02, C05-C09, C13	$CD3^+$ $CD4^+$ $CD8^-$ $CD45RO^+$ $CCR7^+$
	T_{EM}	C10- C12, C14, C46	$CD3^+$ $CD4^+$ $CD8^-$ $CD45RO^+$ $CCR7^-$
γδT cell	γδT cell	C15, C22, C40-C43	$CD3^+$ TCR γ/δ^+
$CD8^+$ T cell	Naïve $CD8^+$ T	C16	$CD3^+$ $CD4^-$ $CD8^+$ $CD45RO^-$ $CCR7^+$
	$CD8^+$ T_{EMRA}	C23-C24, C33-C35, C37	$CD3^+$ $CD4^-$ $CD8^+$ $CD45RO^-$ $CCR7^-$
	$CD8^+$ T_{EM}	C38-C39	$CD3^+$ $CD4^-$ $CD8^+$ $CD45RO^+$ $CCR7^-$
NKT cell	NKT cell	C25, C36	$CD3^+$ TCR γ/δ^- $CD56^+$
NK cell	NK cell	C26-C33	$CD3^-$ $CD19^-$ $CD56^+$
Monocyte	Monocyte	C52-C53	$CD3^-$ $CD19^-$ $CD66b^-$ $CD14^+$ $HLADR^+$
DC	DC	C48-C50, C59	$CD3^-$ $CD19^-$ $CD66b^-$ $CD14^-$ $HLADR^+$
B lymphocyte	B lymphocyte	C54-C58, C60	$CD3^-$ $CD19^+$
other	other	C47, C51	$CD3^-$ $CD19^-$ $CD56^-$ $CD66b^-$ $CD14^-$ $HLADR^-$

Abbreviations: DC = dendritic cells; NK = natural killer; PBMCs = peripheral blood mononuclear cells; T_{CM} = central memory T cells; T_{EM} = effector memory T cells; T_{EMRA} = terminally differentiated effector memory re-expressing CD45RA T cells.

Supplementary Table 2: List of 01-60 cluster definitions for a quick search

Cluster	Lineage	Subpopulation	Cluster	Lineage	Subpopulation
C1	$CD4^+$ T	T_{EM} (Treg)	C31	NK cells	/
C2	$CD4^+$ T	T_{EM} (Treg)	C32	NK cells	/
C3	$CD4^+$ T	Naive	C33	NK cells	/
C4	$CD4^+$ T	Naive	C34	$CD8^+$ T	T_{EMRA}
C5	$CD4^+$ T	T_{CM} (Th1)	C35	$CD8^+$ T	T_{EMRA}
C6	$CD4^+$ T	T_{CM} (Th1)	C36	NKT	/
C7	$CD4^+$ T	T_{CM} (Tfh)	C37	$CD8^+$ T	T_{EMRA}
C8	$CD4^+$ T	T_{CM} (Tfh)	C38	$CD8^+$ T	T_{EM}
C9	$CD4^+$ T	T_{CM} (Tfh)	C39	$CD8^+$ T	T_{EM}
C10	$CD4^+$ T	T_{EM}	C40	gdT	/
C11	$CD4^+$ T	T_{EM}	C41	gdT	/
C12	$CD4^+$ T	T_{EM} (Th2)	C42	gdT	/
C13	$CD4^+$ T	T_{CM} (Th2)	C43	gdT	/
C14	$CD8^+$ T	T_{EM}	C44	NKT	/
C15	gdT	/	C45	NKT	/
C16	$CD8^+$ T	Naive	C46	$CD4^+$ T	T_{EM}
C17	$CD8^+$ T	T_{EM}	C47	other	/
C18	$CD8^+$ T	T_{EM}	C48	DC	pDC
C19	$CD8^+$ T	T_{EM}	C49	DC	cDC
C20	$CD8^+$ T	T_{EM}	C50	DC	cDC
C21	$CD8^+$ T	T_{EM}	C51	other	/
C22	gdT	/	C52	Monocytes	/
C23	$CD8^+$ T	T_{EMRA}	C53	Monocytes	/
C24	$CD8^+$ T	T_{EMRA}	C54	B cells	/
C25	NKT	/	C55	B cells	/
C26	NK cells	/	C56	B cells	/
C27	NK cells	/	C57	B cells	/
C28	NK cells	/	C58	B cells	/
C29	NK cells	/	C59	DC	pDC
C30	NK cells	/	C60	B cells	/

Abbreviations: DC = dendritic cells; NK = natural killer; PBMCs = peripheral blood mononuclear cells; T_{CM} = central memory T cells; T_{EM} = effector memory T cells; T_{EMRA} = terminally differentiated effector memory re-expressing CD45RA T cells.

Supplementary Table 3: Antibodies used for mass cytometry and the surface marker being detected

List	Label	Markers	Clone	Company
1	89Y	CD45	HI30	BioLegend
2	115ln	CD3	UCHT1	Bio Cell
3	141Pr	CD56	NCAM16.2	BD biosciences
4	142Nd	TCR γ/δ	5A6.E9	Thermofisher
5	143Nd	CD196_CCR6	G034E3	BioLegend
6	144Nd	CD14	M5E2	BioLegend
7	145Nd	IgD	IA6-2	BioLegend
8	146Nd	CD123_IL_3R	6H6	BioLegend
9	147Sm	CD85j_ILT2	GHI/75	BioLegend
10	148Nd	CD19	4G7	Bio Cell
11	149Sm	CD25_IL_2R	24212	R&D
12	150Nd	CD274_PD_L1	29E.2A3	BioLegend
13	151Eu	CD278_ICOS	C398.4A	BioLegend
14	152Sm	CD39	A1	BioLegend
15	153Eu	CD27	O323	BioLegend
16	154Sm	CD24	ML5	BioLegend
17	155Gd	CD45RA	HI100	BioLegend
18	156Gd	CD86	Fun-1	BD biosciences
19	157Gd	CD28	CD28.2	BioLegend
20	158Gd	CD197_CCR7	G043H7	BioLegend
21	159Tb	CD11c	BU15	BioLegend
22	160Gd	CD33	WM53	BioLegend
23	161Dy	CD152_CTLA_4	BN13	Bio Cell
24	163Dy	CD161_NKR_P1A	HP-3G10	BioLegend
25	164Dy	CD185_CXCR5	RF8B2	BD biosciences
26	165Ho	CD66b	G10F5	BioLegend
27	166Er	CD183_CXCR3	G025H7	BioLegend
28	167Er	CD94	HP-3D9	BD biosciences
29	168Er	CD57	HNK-1	BioLegend
30	169Tm	CD45RO	UCHL1	BioLegend
31	170Er	CD127_IL_7Ra	A019D5	BioLegend
32	171Yb	CD279_PD_1	EH12.2H7	BioLegend
33	172Yb	CD38	HIT2	BioLegend
34	173Yb	CD194_CCR4	L291H4	BioLegend
35	174Yb	CD20	2H7	BioLegend
36	175Lu	CD16	3G8	BioLegend
37	176Yb	HLA_DR	L243	BioLegend
38	197Au	CD4	RPA-T4	BioLegend
39	198Pt	CD8a	RPA-T8	BioLegend
40	209Bi	CD11b	M1/70	BioLegend

Supplementary Table 4: Clinical information about the EOPD and LOPD patients

Subject	Onset	Gender	Age	drinking or not	smoking or not	family history	medications	Onset with tremor or not	Onset of disease
1	Early	F	47.61	N	N	N	N	Y	Right limb
2	Early	M	40.79	N	N	his brother has the history of PD	N	N	Left foot tremor for 3 months, left hand tremor for 2 months
3	Early	M	39.62	N	N	N	N	Y	left limb
4	Early	M	37.92	N	N	N	N	N	Both lower limbs
5	Early	M	42.21	N	N	N	N	Y	Right upper limb
6	Early	F	43.61	N	N	N	N	N	Right limb
7	Early	F	37.25	N	N	N	N	N	Both lower limbs
8	Early	M	39.19	N	N	N	N	N	Unclear
9	Early	M	40.33	N	smoking history for 20 years but has stopped for 3years	N	N	N	left limb
10	Early	M	34.51	N	N	N	N	N	left limb
11	Late	M	63.52	N	N	his mother has the history of tremor, but not making a definite diagnosis	N	Y	Left upper limb
12	Late	F	63.67	N	N	N	N	N	Left upper limb
13	Late	F	60.66	N	N		N	N	Right upper limb

14	Late	M	59.18	N	N	his father has the history of tremor, but not making a definite diagnosis	N	N	Right limb
15	Late	F	73.77	N	N	N	N	N	Right upper limb
16	Late	M	60.86	N	N	N	N	Y	Right limb
17	Late	M	54.75	N	N	N	N	N	Right upper limb
18	Late	M	61.76	drinking for 40 years	smoking for 40 years	N	N	N	Right limb
19	Late	M	56.13	N	N	N	N	Y	left limb
20	Late	M	78.17	N	N	N	N	Y	Lower right limb
21	Late	M	70.98	N	N	N	N	Y	Left upper limb
22	Late	F	67.36	N	N	N	N	Y	Left upper limb

Abbreviations: EOPD = early-onset PD; LOPD = late-onset PD; M = male; F = female; N = No; Y = Yes.
