# Supplementary material for: Specific immune status in Parkinson’s disease at different ages of onset
Source: NPJ Parkinsons Dis. 2022 Jan 10;8:5. doi: 10.1038/s41531-021-00271-x (PMC8748464; doi:10.1038/s41531-021-00271-x)
Supplement: Supplementary file 2 — Reporting Summary [file 41531_2021_271_MOESM2_ESM.pdf]

nature portfolio

Corresponding author(s): Jia-Li Pu

Last updated by author(s): Aug 12, 2021

Reporting Summary

Nature Portfolio wishes to improve the reproducibility of the work that we publish. This form provides structure for consistency and transparency in reporting. For further information on Nature Portfolio policies, see our Editorial Policies and the Editorial Policy Checklist.

Statistics

For all statistical analyses, confirm that the following items are present in the figure legend, table legend, main text, or Methods section.

n/a	Confirmed	
☐	☒	The exact sample size (*n*) for each experimental group/condition, given as a discrete number and unit of measurement
☐	☒	A statement on whether measurements were taken from distinct samples or whether the same sample was measured repeatedly
☐	☒	The statistical test(s) used AND whether they are one- or two-sided<br>*Only common tests should be described solely by name; describe more complex techniques in the Methods section.*
☒	☐	A description of all covariates tested
☒	☐	A description of any assumptions or corrections, such as tests of normality and adjustment for multiple comparisons
☐	☒	A full description of the statistical parameters including central tendency (e.g. means) or other basic estimates (e.g. regression coefficient) AND variation (e.g. standard deviation) or associated estimates of uncertainty (e.g. confidence intervals)
☐	☒	For null hypothesis testing, the test statistic (e.g. *F*, *t*, *r*) with confidence intervals, effect sizes, degrees of freedom and *P* value noted<br>*Give P values as exact values whenever suitable.*
☒	☐	For Bayesian analysis, information on the choice of priors and Markov chain Monte Carlo settings
☒	☐	For hierarchical and complex designs, identification of the appropriate level for tests and full reporting of outcomes
☐	☒	Estimates of effect sizes (e.g. Cohen's *d*, Pearson's *r*), indicating how they were calculated

Our web collection on statistics for biologists contains articles on many of the points above.

Software and code

Policy information about availability of computer code

Data collection	No softwares were used.
Data analysis	FlowJo, X-shift clustering algorithm, t-distributed stochastic neighbor embedding algorithm, SPSS version 25.0 (Armonk, NY: IBM Corp),GraphPad Prism 9.0.0(GraphPad Software,LCC)

For manuscripts utilizing custom algorithms or software that are central to the research but not yet described in published literature, software must be made available to editors and reviewers. We strongly encourage code deposition in a community repository (e.g. GitHub). See the Nature Portfolio guidelines for submitting code & software for further information.

Data

Policy information about availability of data

All manuscripts must include a data availability statement. This statement should provide the following information, where applicable:

- Accession codes, unique identifiers, or web links for publicly available datasets
- A description of any restrictions on data availability
- For clinical datasets or third party data, please ensure that the statement adheres to our policy

The data that support the findings of this study are not openly available due to human data and are available from the corresponding author upon reasonable request.

Field-specific reporting

Please select the one below that is the best fit for your research. If you are not sure, read the appropriate sections before making your selection.

☒ Life sciences ☐ Behavioural & social sciences ☐ Ecological, evolutionary & environmental sciences

For a reference copy of the document with all sections, see nature.com/documents/nr-reporting-summary-flat.pdf

Life sciences study design

All studies must disclose on these points even when the disclosure is negative.

Sample size	22 patients and 18 healthy were recruited based on prior mass cytometry studies. No sample-size calculation was performed.
Data exclusions	No data exclusions.
Replication	This study did not perform replication experiments.
Randomization	The patients and healthy controls are selected in patients admitted to our hospital or healthy controls we recruited via simple random sampling.
Blinding	Blinding was not relevant to this study because the data were clinical objective index.

Reporting for specific materials, systems and methods

We require information from authors about some types of materials, experimental systems and methods used in many studies. Here, indicate whether each material, system or method listed is relevant to your study. If you are not sure if a list item applies to your research, read the appropriate section before selecting a response.

Materials & experimental systems

n/a	Involved in the study	
☐	☒	Antibodies
☒	☐	Eukaryotic cell lines
☒	☐	Palaeontology and archaeology
☒	☐	Animals and other organisms
☐	☒	Human research participants
☒	☐	Clinical data
☒	☐	Dual use research of concern

Methods

n/a	Involved in the study	
☒	☐	ChIP-seq
☐	☒	Flow cytometry
☒	☐	MRI-based neuroimaging

Antibodies

Antibodies used	The antibodies used are listed in Supplementary Table 3.
Validation	The information of antibodies used are listed in Supplementary Table 3.

Human research participants

Policy information about studies involving human research participants

Population characteristics	Age, gender, duration of disease and clinical evaluation of parkinson's disease via scales were collected (Table 1).
Recruitment	All patients were admitted to the neurology department at the Second Affiliated Hospital of Zhejiang University and diagnosed by senior movement disorder specialists based on the current diagnostic criteria.
Ethics oversight	Ethics approval was obtained through the Medical Ethics Committee of the Second Affiliated Hospital of Zhejiang University School of Medicine (2020-596). All patients and HCs provided their informed consent before blood withdrawal.

Note that full information on the approval of the study protocol must also be provided in the manuscript.

Flow Cytometry

Plots

Confirm that:

☒ The axis labels state the marker and fluorochrome used (e.g. CD4-FITC).

☒ The axis scales are clearly visible. Include numbers along axes only for bottom left plot of group (a 'group' is an analysis of identical markers).

☒ All plots are contour plots with outliers or pseudocolor plots.

☒ A numerical value for number of cells or percentage (with statistics) is provided.

Methodology

Sample preparation	The details of sample preparation were described in method.
Instrument	Beckman Centrifuge with plate rotor; Helios Mass cytometry; ALIT Life Science Countstar
Software	FlowJo, X-shift clustering algorithm, t-distributed stochastic neighbor embedding algorithm
Cell population abundance	N/A – no populations were sorted
Gating strategy	Mass Cytometry<br>After normalization and debarcoding of files, singlets were gated by Event Length and DNA. Live cells were identified by Cisplatin negative cells. Erythrocytes were excluded by gating on Ter119- cells. All positive and negative populations and antibody staining concentrations were determined by titration on positive and negative control cell populations.<br><br>Populations were defined as follows.<br><br>pDCs (CD45+B220+PDCA1+Ly6G-SiglecF-Fcer1a-)<br>B cells (CD45+B220+CD19+CD138-Ly6G-SiglecF-Fcer1a-)<br>CD8 T cells (CD45+CD3+CD8+TCRgd-NK1.1-B220-CD19-CD138-Ly6G-SiglecF-Fcer1a-)<br>CD4 T cells (CD45+CD3+CD4+FoxP3-CD8-TCRgd-NK1.1-B220-CD19-CD138-Ly6G-SiglecF-Fcer1a-) Tregs (CD45+CD3+CD4 +FoxP3+CD8-TCRgd-NK1.1-B220-CD19-CD138-Ly6G-SiglecF-Fcer1a-) gdTCells (CD45+CD3+TCRgd+B220-CD19-CD138-Ly6G-SiglecF-Fcer1a-)<br>NKT Cells (CD45+CD3+NK1.1+TCRgd-B220-CD19-CD138-Ly6G-SiglecF-Fcer1a-)<br>NKT Cells, non-Black6 mice (CD45+CD3+CD49b+TCRgd-B220-CD19-CD138-Ly6G-SiglecF-Fcer1a-)<br>NK Cells (CD45+CD3+NK1.1+CD49b+CD3-CD19-B220-CD138-Ly6G-SiglecF-Fcer1a-)<br>NK Cells, non-Black6 mice (CD45+CD3+CD49b+CD3-CD19-B220-CD138-Ly6G-SiglecF-Fcer1a-) Macrophages (CD45+CD64 +F480+Ly6G-SiglecF-CD19-B220-CD3-Fcer1a-NK1.1-CD138-)<br>cDCs (CD45+CD11c+MHCII+CD64-F480-NK1.1-CD3-CD19-B220-CD138-Fcer1a-SiglecF-Ly6G-)<br>cMonocytes (CD45+CD115+Ly6C+CD11c-CD64-F480-NK1.1-CD3-CD19-B220-CD138-Fcer1a-SiglecF-Ly6G-) ncMonocytes (CD45+CD115+Ly6C-CD64-F480-NK1.1-CD3-CD19-B220-CD138-Fcer1a-SiglecF-Ly6G-)<br>ILCs (CD45+CD90+CD115-CD64-F480-NK1.1-CD3-CD19-B220-CD138-Fcer1a-SiglecF-Ly6G-)<br>Other (CD45+CD115-CD64-F480-NK1.1-CD3-CD19-B220-CD138-Fcer1a-SiglecF-Ly6G-)

☒ Tick this box to confirm that a figure exemplifying the gating strategy is provided in the Supplementary Information.
